# Evaluating case management for caregivers of children with spinal muscular atrophy type I and II—an exploratory, controlled, mixed-methods trial

**DOI:** 10.3389/fped.2023.1212012

**Published:** 2023-09-20

**Authors:** Jana Willems, Astrid Pechmann, Sabine Wider, Rita Ambs, Sylvia A. N. Meyer, Isabel Cascante, Joachim Sproß, Annette Mund, Erik Farin-Glattacker, Thorsten Langer

**Affiliations:** ^1^ Section of Health Care Research and Rehabilitation Research, Institute of Medical Biometry and Statistics, Faculty of Medicine and Medical Center, University of Freiburg, Freiburg, Germany; ^2^ Department of Neuropediatrics and Muscle Disorders, Center for Pediatrics, Faculty of Medicine and Medical Center, University of Freiburg, Freiburg, Germany; ^3^ Children’s Hospital, Klinikum Esslingen, Esslingen am Neckar, Germany; ^4^ Deutsche Gesellschaft für Muskelkranke, Waltershofen, Germany; ^5^ Kindernetzwerk e.V., Berlin, Germany

**Keywords:** case management, rare diseases, spinal muscular atrophy, integrated care, health-related quality of life

## Abstract

**Introduction:**

Spinal muscular atrophy (SMA) is a rare neuromuscular disease requiring various clinical specialists and therapists to provide care. Due to the disease's dynamic nature and the long distances between specialized centers and local providers, integrating care between disciplines can be challenging. Care that is inadequately integrated can compromise the quality of care and become a burden for patients and families. This trial aimed to improve the care of patients through a case management (CM) intervention.

**Methods:**

We conducted an exploratory, controlled, two-arm trial with pre-, post-, and follow-up measures (process and outcome evaluation). Proof of efficacy based on statistical significance was not our primary study objective since we were investigating a rare disease. Primary outcomes were caregivers' HRQoL and caregiver-rated quality of care integration. Our secondary outcome was the children's HRQoL.

**Results:**

Questionnaires and semi-structured interviews yielded heterogeneous results depending on caregivers' level of experience and desire (or possibility) to delegate care tasks.

**Discussion:**

Despite differing perceptions, all participants supported the establishment of a care coordination model. We recommend CM immediately after diagnosis to provide the greatest benefit to families. We hope that our trial will support the further development of CM interventions that can be customized for specific diseases.

## 1. Introduction

Spinal muscular atrophy (SMA) is a rare genetic, neuromuscular disease (incidence of 1:6,000–1:10,000 births/year) characterized by the degeneration of alpha motor neurons in the spinal cord caused by the loss or dysfunction of the SMN-1 gene 5q11-q13 (1). Despite therapeutic advances and significant improvements in pharmacological treatment (2–7), SMA has remained a chronic complex condition for the majority of patients over the past years (8). According to current recommendations, this patient population needs multidisciplinary care (1, 9). There are local care providers near the families’ place of residence (e.g., occupational therapy, physiotherapy, speech therapy, and home care service) and specialized facilities [e.g., neuromuscular center, social pediatric center (SPC), etc.] that provide drug therapy along with other SMA-specific services, and are often located farther away. As the care in many healthcare systems is so fragmented, caregivers spend a lot of time coordinating appointments, communicating between providers, clarifying sociolegal issues, and more (10). These demands, and handling the disease's progressive nature influence the entire family's health-related quality of life (HRQoL) (11–21). Effective care coordination is needed that can flexibly adapt to families’ changing needs. To achieve good care coordination, good collaboration in the care network [= patient/caregivers, healthcare professionals, health insurance companies, and other services (22)] is mandatory (23). In addition, families require up-to-date information, counseling, and psychosocial support to cope with the burdens and treatment decisions that influence the quality and length of their children's lives. The care situation of children with SMA serves as an example for a large group of patients suffering from chronic complex conditions, and reveals the need for comprehensive healthcare service that incorporates families’ participation (24, 25). New care concepts that address the needs of families and optimize the flow of information between neuromuscular centers and local treatment providers are urgently needed.

In this study, we investigated the impact of a Case Management (CM) intervention on the quality of care integration of patients with SMA and caregivers’ and children's HRQoL. “Care integration” includes all aspects of care coordination, but additionally describes a more flexible care system that can respond to the needs of patients and families. It therefore entails patient-centeredness (24) and assesses the quality of coordinated activities from the families’ perspective (26). Good care coordination is a precondition for good care integration. Our CM is a case-oriented interdisciplinary care coordination model that supports families within their child's care management (27). It has been designed to improve the patient-centeredness of care and to empower caregivers in their care management. A fixed contact person within the care network facilitates family-related organization and therefore enables long-term assistance and needs-oriented networking on an individual level (28). This CM model includes comprehensive provision of information, integration between the patient's multidisciplinary healthcare providers, and support for the family, with emphasis on high-quality, cost-effective care (27, 29–33). We conducted a process evaluation of a CM intervention approach tailored to the needs of patients with SMA I and II [the most severe subtypes (1)] and their caregivers.

## 2. Materials and methods

### 2.1. Study design and research aims

This study is part of the SMA-C+ project that began in February 2019 and continued until June 2022 (34). We conducted an exploratory, prospective, controlled, two-armed trial and a process evaluation to assess the impact of a CM intervention on the affected families’ HRQoL and on care integration (intervention group; IG). The control group (CG) received current practice (“usual care”), complying with the standard of care (35). This trial was “exploratory”, since a rare disease was being studied and thus relatively low case numbers were feasible. As with all quantitative data analyses, and within the framework of our study design, one cannot assume that proof of efficacy is possible based on statistical significance tests due to the low case numbers affected by rare diseases.

Our research questions were:

Primary outcomes
  1. How does care integration's quality change after introducing the CM intervention?
  2. How does the caregiver's HRQoL change after introducing the CM intervention?
  3. How does the quality of care integration and HRQoL change in the CG compared to IG?
Secondary outcomes
  4. How does the quality of care and child's HRQoL change after introducing the CM intervention?

Our hypotheses were:

Primary outcomes
  1. The CM intervention leads to better parent-reported care-integration quality.
  2. The CM intervention leads to better HRQoL of caregivers.
  3. Comparing IG and CG, the quality of care integration and HRQoL improved more in the IG.
Secondary outcomes
  4. Relieving the burden on families (through the CM intervention) should lead to an improvement in quality of care and child's HRQoL in IG.

We applied qualitative and quantitative methods (semi-structured interviews and questionnaires) to yield chosen outcomes and evaluate the CM intervention in terms of research questions. We conducted semi-structured interviews and administered questionnaires at the start of intervention (T0), 6 months after the start of intervention (T1), and after a 12-month follow-up period (T2)., the assessment time points were identical in the CG, T0 being the time when the respondents were recruited. Data collection took place between April 2020 and December 2021.

### 2.2. Participants and recruiting

*N* = 32 caregivers (IG: *n* = 21; CG: *n* = 11) whose children have a genetically confirmed SMA type I or type II participated in the study. We recruited participants in the IG at the Department of Neuropediatrics and Muscle Disorders, Freiburg, and CG participants at the Essen Center for Rare Diseases and Department of Child Neurology in Tuebingen. Participation was independent of the length of the child's disease history, i.e., inclusion was possible immediately after diagnosis, or at a later stage. We continuously selected potential participants using a maximum variation sampling approach (purposeful sampling) guided by contrasting characteristics, such as patient age, health condition/stage of illness, family situation, geographic location, and knowledge of German. Treating neuropediatricians at neuromuscular centers recruited participants through personal contact and controlled eligibility criteria during the recruitment process. All caregivers provided written informed consent to participate. Participants in the IG received a fixed sum of 60 € and participants in the CG received 25 € per interview or questionnaire completed.

### 2.3. Development and characteristics of the CM intervention

We created our concept in accordance with established CM models, e.g., CM to support families after discharge from hospital of premature infants (28, 29). CM was located at the neuromuscular center of the University Children's Hospital Freiburg, Germany. Two certified pediatric nurses and certified CM assistants took on the role of case managers in the project. Both case managers had clinical experience with the disease and were well informed about current care options. We held two symposia to which we invited concerned caregivers, chairpersons of patient advocacy organizations, and healthcare professionals involved in the care network. In these symposia, we worked out the CM intervention's individual elements and its overall design. CM was designed to assess each care situation in its entirety together with the families to identify areas of sub-standard care and insufficient care integration. A concrete diagram of our case management's theoretical basis can be found in the study protocol of the SMA-C+ trial (34).

### 2.3. Characteristics of the CM intervention

- Regular, structured conversation with participants regarding overall care including the organization of specialist appointments, provision of medical aids, the child's integration into pre-school and school activities, family support
- Information on (new) care options including informing families about helpful contacts, e.g., a clinic performing spine surgery on children with SMA
- Support in organizing and coordinating appointments within the neuromuscular center, i.e., combining appointments with different specialists to avoid repeated visits to the neuromuscular center
- Supporting information exchanges between providers in and outside the hospital who do not communicate with each other on a regular basis, e.g., physiotherapists

In previously conducted interviews with caregivers of SMA I and II children (multi-perspective state analysis; 17), we obtained information on which SMA-specific aspects play a role in a tailored CM intervention:

- As SMA is a rare condition, caregivers initially have little knowledge about the disease and strong demand for information requiring input from various care providers, especially right after diagnosis.
- SMA manifests early in a child's life and has a severe impact on the patient and family. During this phase, caregivers are need continuity and support. Experienced caregivers, however, may have different needs, e.g. the child's care situation could be medically stable but the transition from one school to another might be psychologically demanding for the patient and family.
- Since the approval of new drugs, caregivers are facing challenging decisions for which they may benefit from counseling by several healthcare providers, i.e. pediatric neurologists, physiotherapists, psychologists, etc. Until now, most children with SMA have been given Nusinersen (Spinraza), which could require a three-day inpatient stay (e.g., due to a longer journey to the neuromuscular center). With Onasemnogen-Abeparvovec (Zolgensma) and Risdiplam (Evrysdi), this approach is changing: Onasemnogen-Abeparvovec requires just one administration, but is preceded by intensive preliminary testing and follow-up care, sometimes requiring inpatient stays. For example, when switching from Nusinersen to oral administration of Risdiplam, it is the caregiver's responsibility to pick up the drug at a pharmacy and administer it at home with the help of regular prescriptions.

### 2.4. Procedures and outcomes

#### 2.4.1. Primary outcome measurement

**Figure 1:**
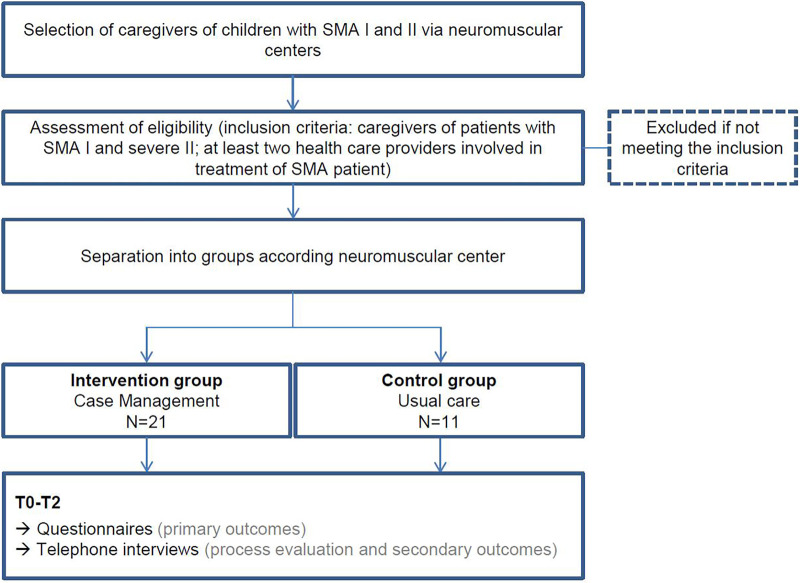
Participant timeline; T0, start of intervention (IG)/recruitment of participants (CG); T1, 6 months after start of intervention/recruitment; T2, 12-months-follow-up-period.

At T0, we sent caregivers paper-pencil pseudonymized questionnaires on primary outcomes (quality of care integration and caregiver's HRQoL) and sociodemographic information. To test for 6–12-month sustainability (T1, T2) caregivers received follow-up questionnaires identical to the T0 measurement. Participant timeline is shown in Figure 1. To examine the impact of the CM intervention on chosen outcomes, we used the following instruments:

1. Quality of care integration from the caregivers’ perspective

In a previous phase of this study, we translated the *Pediatric Integrated Care Survey* questionnaire for German-speaking countries (*PICS-D*) and tested it psychometrically (34). The PICS-D has a three-factorial structure, although only two scales should be used for scoring because the third factor is unreliable. Fit indices suggested a good fit between the model and the data (CFI = 0.95, TLI = 0.94, RMSEA = 0.07). Cronbach's α was 0.89 and 0.84 in factors 1 and 2, respectively. McDonald's ωt. McDonald's ωt was 0.93 and 0.88 in factors 1–2, respectively. The 13-Likert-scaled-item *PICS-D* explores the caregiver-evaluated care integration through two scales: “Team quality & communication” (TQC) and “Family impact” (FI). To assess the intervention's influence on the quality of care integration, we administered the *PICS-D*. Higher scale values indicated better-perceived care integration (36).

2 Caregivers’ HRQoL

We relied on two questionnaires to assess caregivers’ HRQoL:

a. Familien-Belastungs-Fragebogen (FaBel) (37)

We administered the *FaBel* questionnaire to determine how parents themselves assessed the family burden caused by their child's disease. It is based on the *Impact on Family Scale* translated into German and has been psychometrically tested (38). It contains 33 Likert-scaled items to capture the daily social burden of caregivers, the personal burden, the burden of siblings, the financial burden, problems coping with the burden, and a total score (FaBel-T) of the overall burden. Higher scale values indicated a heavier burden.

b. Short-Form-Health-Survey [SF-12, short version of SF-36 (39)]

We administered the SF-12 to assess caregiver-reported health-related quality of life during the past four weeks. This is a generic 12-item questionnaire that supplies a subjective health status summary score of physical (SF12-K) and mental health (SF12-P). This total value results from four health components each: Physical health comprises *general health, physical functioning,* and *role limitations due to physical health problems and bodily pain*; subjective mental health comprises *vitality (energy/fatigue), social functioning,* and *role limitations due to emotional problems and mental health*. Higher scale values indicated better health.

#### 2.4.2. Process evaluation of CM and secondary outcome measurement

1. Process evaluation of CM and assessment of quality of care

We conducted recurring semi-structured telephone interviews with caregivers at the three time points (T0-T2). We asked caregivers about their assessments of care integration at T0, and about changes over time (T1/T2). Furthermore, we gathered information on the child's unplanned medical and therapeutic care and the caregiver's work absenteeism (to provide child's care). In the IG, telephone interviews also functioned to evaluate the CM intervention process (perception of the intervention's feasibility concerning care integration). The telephone interview lasted on average 15 min and was conducted by a psychologist on the project team. The semi-structured interview guides were drafted by T.L. and J.W. following Helfferich (40) and finalized after review by the whole team. We adjusted the interview guides slightly between measurement time points to account for recent events in the care of children with SMA (e.g., COVID-19, new drug approvals). All interviews were digitally audiotaped in full after having gotten permission from the participants. The audio recordings were transcribed verbatim by an external transcription service provider and personal data was pseudonymized.

2. Child's HRQoL

In addition, the child's HRQoL was assessed by caregivers using the PedsQL™ 4.0 SF15 (41). The Likert-scaled 15-item questionnaire is a shortened version of the 23-item PedsQL™ 4.0 Generic Core Scales that was developed to measure generic pediatric health-related quality of life over the past 4 weeks. We used the adult report standard versions for toddlers (2–4 years of age) and young children (5–7 years of age) as the participating SMA patients were all within this age range. The questionnaire contains four scales addressing problems in physical functioning, emotional functioning, social functioning, school functioning, and a total score (PedsQL-T).

An overview of all implemented questionnaires (with example items) is found in Table 1.

**Table 1:** Questionnaire scales and example items related to chosen outcomes.

Outcome	Questionnaire	Scales of interest
Quality of care integration	PICS-D
	Example item: *How often did you feel that treatment recommendations were passed between the members of the care network?*	Team quality & communication Family impact
Caregivers’ HRQoL	**FaBel** Example item: *Because of my child's illness, I am constantly overtired and fatigued*.	Total score
	**SF-12** Example item: *During the past 4 weeks, how much of the time has your physical health or emotional problems interfered with your social activities (like visiting friends, relatives, etc.)?*	Physical Health Mental Health
Child's HRQoL	**PedsQL SF15** Example item: *In the past one month, how much of a problem has your child had playing with other children?*	Total score

### 2.5. Data management and analysis

Participants sent the completed questionnaires directly to the evaluating institute. Certified research assistants and project coordinators entered the data. Standard processes (i.e., verifying that data is within an expected range of values) were implemented to improve the accuracy of data entry. Questionnaires were digitalized. The research data (pseudonymized transcripts, questionnaire data) were stored digitally on the secure, restricted-access project drive of the evaluating institute. Personal data were kept separate from scientific data, only accessible to selective project coordinators, and will be deleted 36 months after the study's end.

#### 2.5.1. Statistical analyses

##### 2.5.1.1. Quantitative analyses

We performed all quantitative statistical analyses using the Software R Version 4.0.3 (42). We used linear mixed models (LMM) with random intercepts to examine the impact of group (IG, CG) and time point (T0–T2) as well as potential cross-level interaction effects on the chosen outcomes. The cluster variable was the case ID (level 2 measurement) with the time point nested within it (level 1 measurement). We performed separate analyses for all criteria (scales of the questionnaires used). Because we were investigating a rare disease, we were unable to enroll enough participants to test for statistical significance. Instead, our analyses focused on determining effect sizes [ES; marginal R^2^, R^2^m (43)]. R^2^m is the proportion of variance in the dependent variable explained by the mean differences in all fixed effects (predictors = group and time point). We tested the hypothesis of whether the ES for all analyses would be at least medium according to usual conventions (R^2^m ≥ 0.06). We performed analyses with the packages lme4 (44) and lmerTest (45). We used Satterthwaite's degrees of freedom implemented in the package lmerTest.

##### 2.5.1.2. Qualitative analyses

We analyzed the transcripts of the interviews via qualitative content analysis largely based on the Kuckartz approach (46). We took a deductive-inductive approach to create a code system. To ensure intersubjective comprehensibility, the whole team reviewed the coding frame and guide. The multi-level procedure chosen for our study is outlined below: (a) We read intensively the transcribed text material in the process of pseudonymization and composed short case summaries. (b) We extracted codes inductively using the case summaries. In the next step, additional codes were derived deductively from key topics of the interview guides and previous research on secondary outcomes. (c) We applied this initial coding frame to a quota sample consisting of 20% of the data material (*N* ≈ 7). We only applied the process evaluation codes concerning the CM intervention feasibility on IG-transcripts. During this process, all codes were refined several times through continuous reflection, and classified into main and sub-codes. (d) We then started the first coding of the entire material along with the so-far-defined coding frame. Codes were revised again if required, e.g., summarized or differentiated into further sub-codes. We discussed discrepancies and defined the final set of three main codes and eight sub-codes by consensus (for an overview see Table 2). (f) J.W. applied this final coding system applied to all transcripts. This process of (sub-) code formation was iterative until acceptable discriminatory power and depth of categories were achieved. (g) In the next step, we paraphrased all statements of a participant assigned to the same code. Overall findings were extracted from a code × participant-summary-matrix separated according groups (IG, CG). Data was organized and analyzed using the qualitative data analysis software MAXQDA Plus 2020 (version 20.0.3). We conducted the interviews in German. We translated code descriptions and quotations taken from the transcript into English.

**Table 2:** Final coding system assessment of care integration and CM process evaluation.

Main codes	Sub-codes
Care integration at T0	Information on SMA
	Care network
Process evaluation of CM intervention (IG only)	CM topics
	Contact with case manager
	Overall assessment
	Desired CM elements
Changes in care integration (T1/T2)	Changes in information on SMA
	Changes in care network

###### 2.5.1.2.1. Coding system

This section is intended to provide a brief overview of the coding system (Table 2). Because we sought a group comparison in terms of interview statements and only IG participants received CM, the CM codes apply only to interview statements made by IG participants and represent a process evaluation of the CM intervention assessed by the IG's caregivers. “CM topics” describes reported issues discussed with the case managers. “Contact with case manager” describes the families’ contact with case managers. It concerns both the perception of the contact by the caregivers and the design of the contact framework (e.g., frequency of contact, type of contact, etc.). “Overall assessment” refers to an overall assessment of the CM intervention in terms of feasibility, acceptance, usefulness (relief), and integration into the caregiver's care management. “Desired CM elements” describes suggestions made by caregivers to further develop the CM intervention.

We assessed the status quo of care integration at T0. At that time, the CM intervention in the IG was just starting, so the code “Care integration at T0” and its sub-codes could provide an overview of the care situation of children with SMA from the caregivers’ perspective. Therefore, it applies equally to both groups. The sub-code “Information on SMA” describes the methods and channels through which participants obtained information about SMA. Regarding the sub-code “Care network”, participants provided information on the characteristics of their care network (= all healthcare professionals involved in the child's care). In addition to the process evaluative codes for IG caregivers’ assessment of the CM intervention, we chose the code “(Change in) Care integration” that captures changes in care integration from the caregivers’ perspective in both groups over measurement time points. The two selected sub-codes include key aspects targeted by the CM intervention (CM as a potential source of information; case manager as an ongoing contact in the care network).

## 3. Results and findings

As already stated in section “2.1. Study design”, proof of efficacy based on statistical significance tests was not this study's primary objective. For the purpose of completeness, we nevertheless report results of significance tests.

### 3.1. Sample

Detailed child and caregiver descriptive characteristics at T0 separated by group are in Table 3.

**Table 3:** Child and caregiver descriptive characteristics at T0 separated by group.

Variable	IG (*n* = 21)	CG (*n* = 11)
Respondent gender		
Female	17 (81.0)	2 (18.2)
Male	4 (19.0)	9 (81.8)
Mean respondent age at questionnaire completion (SD)	34.6 (5.2)	38.4 (4.7)
Respondent education		
Primary school, secondary school/secondary modern	5 (23.8)	–
High school	3 (14.3)	1 (9.1)
Completed training	7 (33.3)	2 (18.2)
University degree (Bachelor, Master, Doctorate)	5 (23.9)	4 (36.4)
Other	1 (4.8)	4 (36.4)
Respondent family status		
Single	3 (14.3)	–
Married	15 (71.4)	10 (90.9)
Living in a steady partnership	2 (9.5)	1 (9.1)
Divorced, separated	1 (4.8)	–
Respondent employment status		
Employee full-time	4 (19.0)	2 (18.2)
Employee part-time	9 (42.8)	4 (36.4)
Civil servant	–	1 (9.1)
Self-employed	1 (4.8)	–
Not gainfully employed or capable of gainful employment	4 (19.0)	3 (27.3)
Other	2 (9.5)	–
Work absenteeism (to provide child's care)	4 (19.0)	3 (27.3)
Child gender		
Female	9 (42.9)	6 (54.5)
Male	12 (57.1)	5 (45.5)
Child age at questionnaire completion		
1–3 years	7 (33.3)	3 (27.3)
3–12 years	13 (61.9)	7 (63.6)
12–18 years	1 (4.8)	1 (9.1)
Mean time between diagnosis and T0-interview in years (SD)	3.79 (2.22)	4.82 (3.44)
Number of healthcare providers involved in child's care		
2–5	7 (33.3)	1 (9.1)
6–10	9 (42.9)	7 (63.6)
11–15	1 (4.8)	2 (18.2)
>15	2 (9.5)	1 (9.1)
Health insurance		
Statutory	16 (76.2)	10 (90.9)
Statutory with additional insurance	4 (19.0)	–
Private	–	1 (9.1)
Unplanned medical and therapeutic care	7 (33.3)	5 (45.5)
Primary language spoken at home		
German	13 (61.6)	10 (90.9)
Other	7 (38.4)	1 (9.1)

Not all participants completed all questions. All differences between groups were non-significant.

At T0, 21 caregivers participated in the IG, at T1 the number of participants dropped to *n* = 17, and by T2, 20 caregivers were participating. In the CG, the number of participants was *n* = 11 at each time point. There was an increase in IG participants from T1 to T2 because we were able to motivate three of the caregivers who were already participating at T0 to participate again in the study at T2. The caregivers received CM continuously, but were not able to complete the questionnaires and participate in an interview at T1 because they lacked the time resources.

### 3.2. Primary outcome measurement

1. Quality of care integration from the caregivers’ perspective

**Figure 2:**
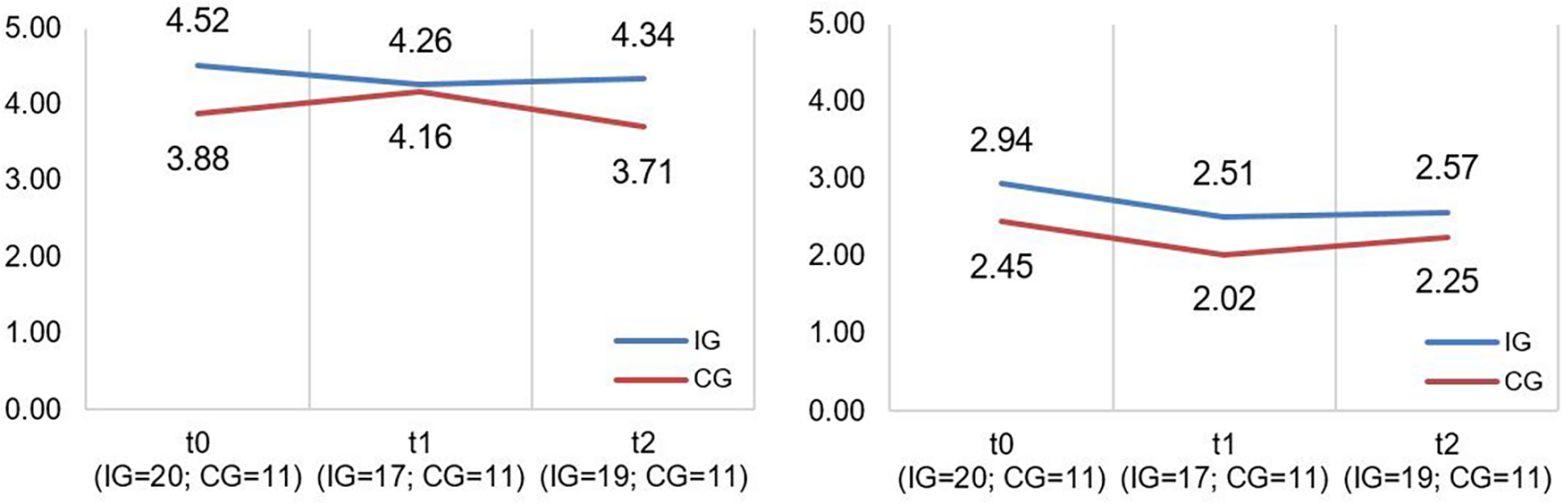
Illustration of mean values in IG and CG for *PICS-D scales* “*team quality and communication*” and “*family impact*” (f.l.t.r.); scale range 1−6; higher scores indicate better perceived care network quality and communication (“*Team quality and communication*”*)*/better perceived communication of disease's impact on the family by the HCP (“*Family impact*”).

A descriptive overview of the scale values for all time points, separated by group, is found in Table 4. A graph of the mean values at all measurement points, separated by group, is found in Figure 2. LMMs failed to yield significant results for the PICS-D-scales “Team quality & communication” and “Family impact”. Relevant parameters (unstandardized regression coefficients with standard errors) are found in Table 5. Targeted medium ES according to usual conventions (R^2^m ≥ 0.06) was achieved for the PICS-D scale “Team quality & communication”.

2. Caregivers’ HRQoL

**Table 4:** Values for PICS-D scales separated by groups and measurement time points.

	Team quality & communication						Family impact					
	IG			CG			IG			CG		
	T0	T1	T2	T0	T1	T2	T0	T1	T2	T0	T1	T2
*n*	20	17	19	11	11	11	20	17	19	11	11	11
M (SD)	4.52 (1.02)	4.26 (0.98)	4.34 (0.91)	3.88 (1.16)	4.16 (1.06)	3.71 (1.31)	2.94 (1.01)	2.51 (0.96)	2.57 (1.17)	2.45 (1.65)	2.02 (1.32)	2.25 (1.39)
min	2.14	1.86	2.00	2.43	3.00	2.43	1.50	1.25	1.00	1.00	1.00	1.25
max	5.86	5.29	6.00	6.00	6.00	6.00	4.75	4.50	5.50	6.00	5.50	5.25

**Table 5:** Multilevel regression results for primary and secondary quantitative outcomes.

	Primary outcomes					Secondary outcome
	Quality of care integration		Caregivers’ HRQoL			Child's HRQoL
	PICSD-TQC	PICSD-FI	FaBel-T	SF12-K	SF12-P	PedsQL-T
Fixed effects						
Intercept	4.52 (0.24)	2.94 (0.27)	8.50 (0.51)	46.09 (2.58)	39.64 (2.86)	44.78 (3.03)
Group (CG)	−0.64 (0.40)	−0.48 (0.45)	0.82 (0.86)	−4.50 (4.40)	−3.24 (4.87)	−1.55 (5.12)
Time (T1)	−0.36 (0.20)	−0.44 (0.19)	0.02 (0.36)	−3.57 (1.86)	0.14 (2.29)	0.34 (2.53)
Time (T2)	−0.15 (0.20)	−0.36 (0.18)	−0.33 (0.33)	−2.84 (1.75)	−0.79 (2.16)	0.12 (2.36)
CG*T1	0.63 (0.33)	0.01 (0.31)	0.26 (0.58)	1.41 (3.01)	0.33 (3.71)	−0.06 (4.05)
CG*T2	−0.02 (0.33)	0.16 (0.31)	0.55 (0.56)	1.80 (2.95)	−4.65 (3.64)	−1.29 (3.94)
Random effects						
Intercept	0.77	1.13	4.24	108.32	124.3	128.62
Residual	0.37	0.33	1.13	30.98	47.2	51.23
R^2^m	0.06	0.05	0.05	0.03	0.04	0.01

PICSD-TQC, PICS-D scale “Team quality & communication”; PICSD-FI, PICS-D scale “Family Impact”; FaBel-T, FaBel total score; SF12-K, SF-12 scale “Physical Health”; SF12-P, SF-12 scale “Mental Health”. Unstandardized regression coefficients with standard errors in parenthesis.

**Figure 3:**
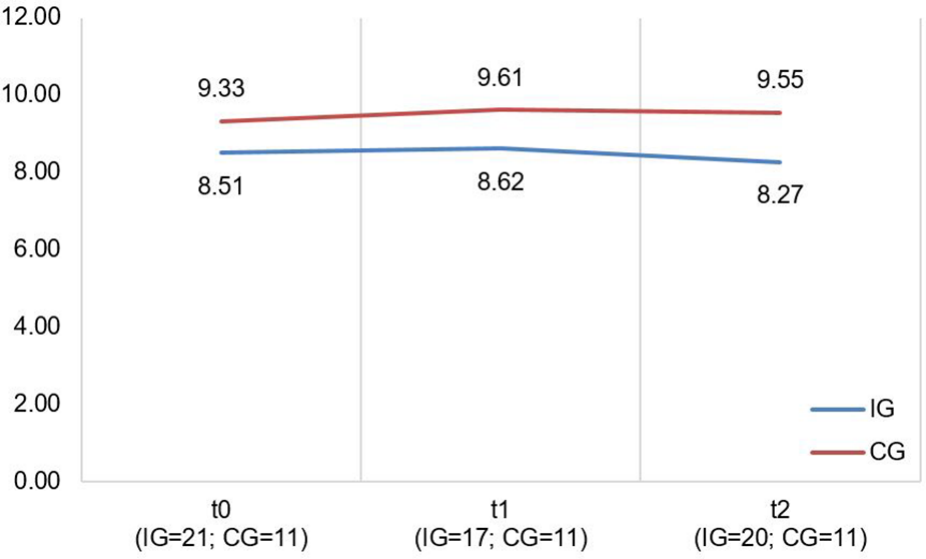
Illustration of mean values in IG and CG for *faBel total score*; scale range 4−16, higher scores indicate higher burden.

**Figure 4:**
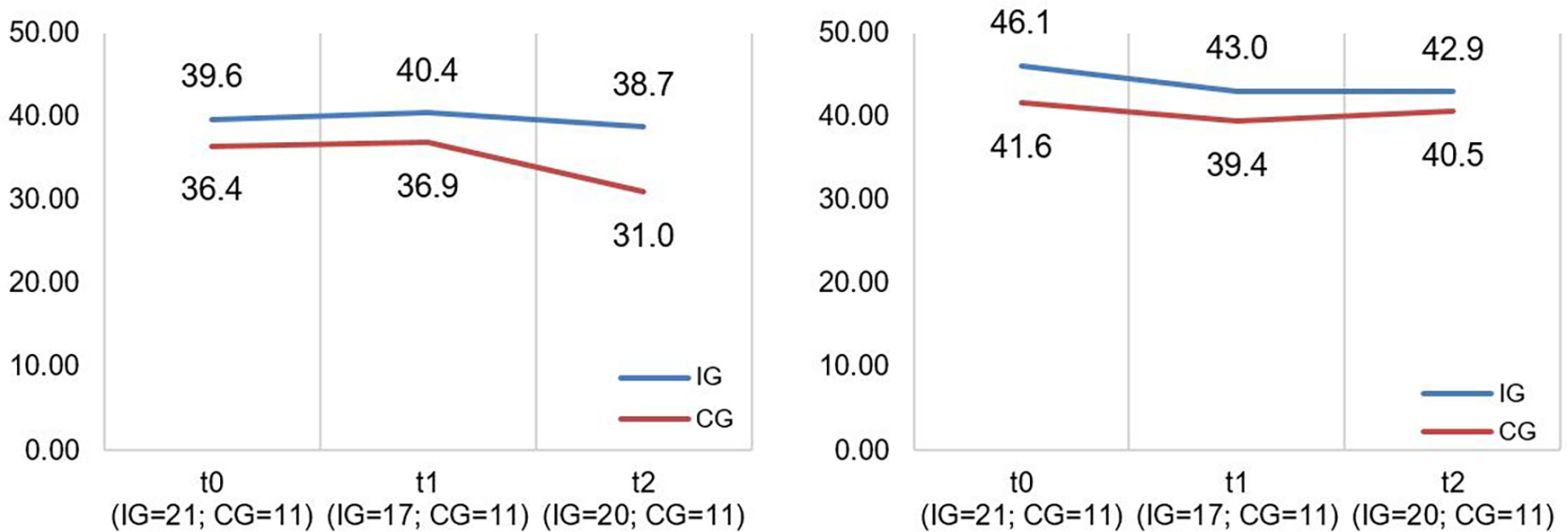
Illustration of mean values in IG and CG for *SF-12 scales* “*mental health*” and “*physical health*” (f.l.t.r.); scale range 0−100; higher scores indicate better mental/physical functioning.

A descriptive overview of the scale values at all time points, separated by group, is found in Tables 6, 7. A graph of the mean values at all measurement points, separated by group, is found in Figures 3, 4. LMMs failed to yield significant results for FaBel- and SF-12 scales. Relevant parameters (unstandardized regression coefficients with standard errors) are found in Table 5. We failed to achieve targeted effect sizes for all scales.

**Table 6:** Values for FaBel total score separated by groups and measurement time points.

	FaBel total score					
	IG			CG		
	T0	T1	T2	T0	T1	T2
*n*	21	17	20	11	11	11
M (SD)	8.51 (1.96)	8.62 (1.83)	8.27 (2.87)	9.33 (2.34)	9.61 (2.18)	9.55 (2.23)
min	3.42	5.65	1.75	5.70	5.75	5.58
max	11.62	11.53	11.93	12.83	12.67	12.60

**Table 7:** Values for SF-12 scales separated by groups and measurement time points.

	Physical health						Mental health					
	IG			CG			IG			CG		
	T0	T1	T2	T0	T1	T2	T0	T1	T2	T0	T1	T2
*n*	21	17	20	11	11	11	21	17	20	11	11	11
M (SD)	46.09 (11.04)	43.01 (11.87)	42.91 (12.11)	41.59 (13.58)	39.43 (12.51)	40.54 (11.06)	39.64 (11.35)	40.43 (12.84)	38.69 (12.91)	36.40 (14.98)	36.88 (15.07)	30.96 (14.00)
min	18.80	18.79	18.79	16.09	21.37	23.80	22.05	18.94	10.09	15.83	15.39	15.39
max	59.08	56.43	59.08	56.37	56.37	53.67	53.83	52.07	54.32	56.52	56.52	54.32

### 3.3. Process evaluation of CM and secondary outcome measurement

1. Process evaluation of CM and assessment of quality of care

Each interview lasted between 3 and 25 min. Child's unplanned medical and therapeutic care and the caregiver's work absenteeism (to provide child's care) at T0 separated by group are found in Table 3.

#### Care integration at T0

Caregivers reported extensive coordination work. This included making care appointments, trying out new care options, getting (second) opinions on new care options, and bringing these options within the child's care network for joint discussion.

*“It was just very, very time-consuming for us to go to several hospitals and listen to their opinions. I think we now have four opinions.”*

##### Sub-code: information on SMA

They obtained information about SMA on their own initiative (e.g., in exchanges with affected caregivers, patient organizations, and social media). New care options would usually be discussed with other affected caregivers first and healthcare professionals second.

*“I first ask for the details in the [WhatsApp] group itself. And then, I actually turn to my contact person at the hospital.”*

##### Sub-code: care network

Caregivers reported difficulties in establishing a care network as well as a lack of care continuity. Furthermore, there was seldom sufficient networking in existing care networks as well as deficient communication. Caregivers stated that it is often their role to enable exchanges within the care network.

*“Everyone does their own tasks, but the cooperation doesn't really work. So since the diagnosis, it's been our turn to somehow build a network, but there's been no chance to do so.”*

Only in rare cases had there been any central coordination point in the care network. Often, individual, informal solutions existed depending on the care network's quality and the initiative of the person who (voluntarily) took over the coordination role (e.g., pediatrician, SPC manager). As care was so fragmented caregivers often had to recruit many health care contacts to meet individual needs.

*“Because there is no one person who pulls the strings and coordinates everything—who I can contact no matter what it’s about, whether it is osteopathy or a prescription for medical aids or anything else—it never goes through a central office, and much of this is simply done by me, because I decide myself to take it into my own hands and simply give it a try.”*

#### Process evaluation of the CM intervention (IG only)

##### Sub-code: CM topics

Reported CM issues appeared to be independent of the time of measurement. Rather, the topics tended to vary among caregivers interviewed, and remain stable over time.

The most frequently mentioned CM topic was the coordination of regular care appointments among various hospital departments. The caregivers emphasized efficient consolidation of appointments into a short period by the case manager to avoid repeated visits to the neuromuscular center (e.g., during a planned inpatient stay for drug administration). They reported that the case manager reminded the departments and themselves of the scheduled appointments. This provided an “automated process” that caregivers could rely on, without having to take action themselves. Furthermore, the case manager enabled flexible rescheduling of appointments.

*“It all goes very quickly with the case manager. She coordinates all our appointments very well so that we do not have to go to the neuromuscular center twice or have too many appointments on one day.”*

Caregivers reported getting assistance with organizing aids, changing home care providers/attending hospitals, parent-child cures, and that the case manager organized prescriptions or x-rays on her own. Furthermore, CM assisted in the establishment and expansion of the (local) care network by providing contact referrals.

*“I also use case management for prescriptions and things like that. She organizes prescriptions and sends them to me, or even x-rays from the orthopedist—I need not worry about that anymore! I write her an e-mail, or we discuss it on the phone, and then she actually takes care of everything.”*

In addition, caregivers described a mediation role for the case manager. Through her medical competence, the caregivers felt supported in their exchange with healthcare professionals or the health insurance company. If they had inquiries to specific physicians of individual departments, the case manager forwarded them and subsequently informed them of the corresponding response. The case manager played a supportive role in communicating with health insurance providers or government agencies. She handled letters of recommendation, helped with declarations of objection, and sometimes even communicated independently with authorities.

*“I think it is positive that we do not always stand in-between as a mediator. Because it is better for medical colleagues to talk to each other than for me to talk to them—as a mother and as a layperson in terms of the technical terms—to stand in between.”*

Furthermore, the caregivers described CM's accompaniment function. The case manager inquired about the family's well-being and living situation during regular telephone calls and found out about the status quo in the child's therapies, support, and relief needs.

*“We also discuss the general situation, how we’re doing personally and what's bothering our child. So we work on such topics during the phone calls.”*

CM also served as an information source. Caregivers received information about new care options or sociolegal issues that they could discuss directly with the case manager.

*“Risdiplam is now approved. So we have now also told the case manager that we would definitely like to have a conversation with Dr. L. about what this change to Risdiplam would mean.”*

##### Sub-code: contact with case manager

During the T0 and T1 interviews, caregivers described a “start-up” period with CM. The case manager and caregiver used this phase to clarify expectations to personalize CM. This involved a detailed assessment to learn about the status quo of the child's care and to determine needs.

*“The first meeting with the case manager was positive. The situation is new for all of us, and we all have to get used to the idea that we can now get started.”*

Caregivers reported heterogeneous contact patterns. While some families favored regularly scheduled conversations at personally set frequencies (e.g., once a month, every 2–6 months), there were likewise caregivers who used CM more on an as-needed basis. However, we also noted a hybrid version of regular appointments and reporting when needed, which was described as “staying in touch permanently”. The most preferred contact channel was via telephone or e-mail, with face-to-face meetings occurring less frequently (e.g., during a regular inpatient stay).

*“We discuss the date for the next phone call each time, in about six weeks. But if I have a question in between, I write her immediately and she answers the same day. So we have appointments for the phone calls and if something comes up in between, then we discuss that too.”*

At T2, we observed a change in the frequency of contact due to new drug approvals. Especially in a new drug's initial phase, there was high demand for information on the part of the caregivers, and medical queries on the part of the neuromuscular center.

*“At the beginning, we occasionally had a few other questions for the case manager, because the new drug was something completely different, something completely new. Particularly in the area of clarifying side effects, for example, or then there were occasional queries about the University Hospital in Freiburg about what the compatibility with other drugs looks like, especially antibiotics, and stories like that. So the contact is a bit more frequent than in Spinraza times.”*

Consistently across all measurement points, caregivers described the contact with the case manager as understanding, always available, and efficient. Requested topics were adequately and completely addressed, and care options were explained in an understandable manner.

*“The case manager can understand how we feel and I can call her at any time and never disturb her—that’s important for us caregivers.”*

##### Sub-code: overall assessment

Because the CM intervention recently started at T0, the results of T0 and T1 assessments were relatively similar and are presented together. Most caregivers described the case manager as a steady and competent contact person who “organizes in the background” and who can be consulted “with any problem”. Thanks to CM, the caregivers would have an individualized care organization characterized by immediacy and reliability in feedback. They reported a lower workload within the care organization by being able to delegate tasks. The case manager would provide continuity in the care network amid a high turnover of healthcare professionals at the neuromuscular center.

*“I think it's good that I only have one contact person who then takes care of the rest. Within 2–3 days a solution was found and what I wanted, I got. With the case manager, everything works very quickly.”*

Caregivers using CM less regularly described a sense of security from the mere existence of a care coordination model. Through CM, they would have the opportunity for regular exchange so that emerging issues in dynamic care could be discussed in a timely manner. By offering an open conversation upon request, caregivers report that they did not feel abandoned amid many different care options.

*“I rely on CM as an exchange to make sure I’m on a good path. Whether the case manager can always help me or not; at least I get an answer. I update them on the status of care and feel like there's someone there who takes you by the hand.”*

However, some caregivers reported that they already had an established network of care in which there was good interaction between them and healthcare professionals. They described themselves as already experienced and accustomed to managing the organization of care for the most part on their own. Other caregivers already had contact persons at other institutions, e.g., the SPC or early intervention center. For these reasons, they did not experience a significant influence on care integration through CM. Some caregivers suggested that they would have found CM to be more supportive immediately after diagnosis.

*“So far, there have been no fundamental changes in care. That is because of our routine. I mean, we have been dealing with the disease for four years now and you have your therapists, your doctors, your regular appointments.”*

Caregivers whose homes were farther away from the neuromuscular center in Freiburg, Germany (where CM was located), reported that CM did not reach into their local network of care close to home. They stated that the benefit was probably greatest if most of care was provided in the area.

*“I always think about whether it is feasible from a distance, with the case management, with the support from Freiburg to our place of residence. You can achieve a lot by phone, but you often have to appear in person.”*

One interviewee pointed out that he perceived CM as still being very theoretical, abstract, and intangible. Furthermore, caregivers reported that they could not or would not delegate certain organizational or coordination aspects of care to the case manager (e.g., longer-term, more extensive projects, such as planning a barrier-free home).

*“I haven't yet perceived that I might somehow draw a benefit from it. For me, CM is still very theoretical. I can't pin it down yet. I wouldn't really say that there is an additional cog intervening in the system and somehow depriving us of areas of activity.”*

In the T2 interviews, most caregivers reported more attuned handling with CM overall. They integrated the case manager into their own care management as a fixed contact person. Due to CM's official availability, the majority of the caregivers experienced more systematic care. They described that the case manager primarily initiated the arrangement of regular check-up appointments (e.g., regular spine x-rays to prevent scoliosis).

*“The case manager and I make one phone call just before the regular inpatient stay to clarify what appointments are needed—we have developed a schedule, and we go over it.”*

Caregivers noted that new drug approvals changed the CM organization. Due to the potential higher frequency of inpatient stays at the beginning of the drug changeover; some families had more intensive contact with the treating physicians. They could clarify individual questions directly on site. In some cases, this resulted in a less extensive use of CM by some caregivers. Nonetheless, caregivers continued to use CM as a means of contact for questions between inpatient stays.

*“He then has mainly outpatient appointments because of the new drug. Therefore, I think it's very important to have CM. If you have a question between outpatient appointments, you can ask it. It is great that you need not wait until a potential outpatient appointment in three months.”*

However, it became even more evident at T2 that not all caregivers rated CM as equally helpful.

*“We still had to do many things ourselves. Making contacts or filling out forms… really taking care of the matter. The work, that is, if you need something from the health insurance companies or from the district office, still remains with the patient himself.”*

##### Sub-code: desired CM elements

Caregivers described two major components that they felt would improve or complete CM: These include a more timely and intensive information initiative about concomitant symptoms of SMA (e.g., scoliosis). In connection with this, the caregivers wished for regular supervision of the care needs by CM and the prophylactic offering of suitable care options. CM should ideally submit the pros and cons of care options (e.g., assistive devices), provide helpful contact recommendations (e.g., spine surgery hospitals), and educate about rights (e.g., ability to take time off work to attend inpatient stays) and responsibilities (e.g., obtain approvals early). As a result, caregivers would be less reliant on information from other more unreliable sources (e.g., other caregivers, the internet). Furthermore, caregivers noted that complementing CM with an easily accessible bundle of information (e.g., a guide, overview brochure, or app that displays the latest research) would be useful within their care organization.

*“You have to research everything yourself in terms of drugs and aids. Maybe you get information through contact with other families, but nothing comes from the doctor. It would be good if CM could monitor the need for care and preemptively let me know what the next step should be. I often had the feeling that many things should have taken place earlier. For example: “Now a walker would be good.” And then the case manager would give me recommendations on which medical supply store would be good for this.”*

The second major component concerns the stimulation of networking within the individual care network. The caregivers would like to see more cooperation and communication among the individual actors. They proposed that it could be the case manager's role to coordinate and clarify responsibilities. Especially a link between CM and local care network (local therapists, pediatrician) would be useful. The case manager should be a contact person with disease-specific knowledge for the local therapists. In this way, caregivers hoped to better cover potential gaps in care.

*“I would like to see feedback between the case manager and pediatrician—that they simply get in touch with each other. Our health insurance company, for example, always writes to our pediatrician if they have any questions about prescriptions. Or, for example, if there's an objection, our pediatrician can perhaps simply consult with case management.”*

#### Change in care integration (T1/T2)

We assessed changes in care integration across the measurement time points using the T1 and T2 interviews. The focus here is on changes that affected CG and IG equally. The changes reported as a result of CM are primarily reflected in the process evaluation codes above.

In the T1 interviews, participants in both groups reported COVID-19-related changes in care integration. Due to the discontinuation of therapies in kindergarten and at school, caregivers had to perform therapeutic applications at home themselves. Inpatient stays changed because only one parent was allowed to be present.

*“In the beginning, we noticed few changes due to the pandemic. N. was still able to attend kindergarten because he was a special needs child and continued to receive the appropriate treatments such as occupational therapy, speech therapy, and physiotherapy. Now he has to stay at home. The situation is much harder for me because I have to compensate for all these applications, which have now been discontinued.”*

In the T2 interviews, caregivers in both groups reported changes in care integration due to the approval of new drugs. In the beginning, these often entailed more frequent inpatient stays. However, in the long term, this meant fewer and shorter inpatient stays overall. Regular care appointments (e.g., orthopedic examinations at the neuromuscular center) often had to be scheduled within a shorter period. Some CG participants reported that these examinations often fell on single days, which was associated with more trips to the neuromuscular center.

*“Because otherwise, everything was always associated with the hospital stay for Spinraza administration. You went there, got an on-site physiotherapy check, got a lung test on-site and the heart muscles were examined. All that now falls on very badly scheduled individual days. That means we drive 120 kilometers there and back. And we have to hope that all the appointments take place on one day and that no emergency occurs, otherwise we usually have to go back again during the same week.”*

Participants in the IG, on the other hand, reported efficient coordination of care appointments with the help of the case manager in terms of changing frequencies of inpatient stays.

*“The case manager schedules the appointments so that we do not have to come to the neuromuscular center and that, in the best case; everything takes place on the same day. This is very beneficial for us because it is a long journey every time.”*

Furthermore, the caregivers in both groups described planned next steps of care due to the child's development (e.g., entry/change in kindergarten/school) and disease progression (e.g., adjustment of aids to current health status; magnetic rods for flexible spinal fusion; need for a school escort).

*“My son is graduating from secondary school this year and will then change schools. We’re already preparing intensively for this, and we have informed ourselves in advance about the possibilities. We’re now clarifying who's responsible for this, what options there are concerning school support, and are in contact with the new school. We need about a year to organize this in advance.”*

##### Sub-code: changes in information on SMA

Participants in both groups reported little change in their strategy for obtaining information about SMA. In the T1 interviews, CG participants discussed Zoom webinars as a result of the COVID-19 pandemic's restrictions. These webinars were offered by neuromuscular centers and supported by the German Society for Muscular Dystrophy. One interviewee emphasized the high information content of these webinars and the efficiency thanks to their accessibility from home. However, at the time of the T2 interview, these seminars were no longer taking place.

*“Nothing has fundamentally changed in that respect. If I need information, I take care of it myself, so no one has ever come to me and presented me with any information.”*

##### Sub-code: changes in care network

Participants in both groups reported regular changes in the care network due to a growing need for more symptomatic treatments (e.g., speech therapists, occupational therapists) or a change in the primary attending hospital.

*“A few new orthotists have joined us, all pretty far away from where we live as well. They are specialized in certain orthoses. So for the corset, we go to Z. about 80 km away, for the ring orthoses also about 80/90 km again somewhere else.”*

In T2, interviewees described increasing complexity of the disease mainly due to new drug approvals.

CG participants reported a consistent lack of networking, continuity, and collaboration, as well as no contact person within the care network.

*“Nothing's changed. It's still exactly the same—everything's in our hands. There's no contact person who somehow keeps us up to date on various things. There are always only these individual contacts. But getting news about SMA or about therapy methods, that's still up to us. Every time we go to the hospital, we have to try to get in touch with the people in charge and ask them.”*

In the IG, CM was able to buffer the uncertainty caused by the potential switch to a new medication as well as strengthen the caregivers in their care management regarding the new challenges (e.g., personal responsibility in terms of Risdiplam organization).

*“Things have improved, it's become easier. I have a contact person who's the case manager. I send her my e-mail and she takes care of everything. Not: I now have to send an e-mail to Dr. T. and to Professor Dr. F., or as the case may be. So I have one contact person who takes care of everything for me.”*

2. Child's HRQoL

**Figure 5:**
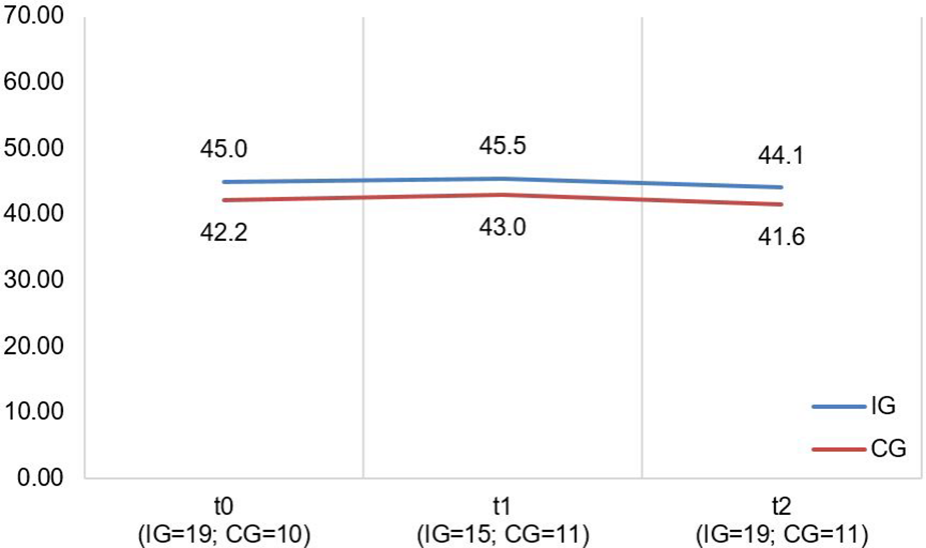
Illustration of mean values in IG and CG for *pedsQL total score*; scale range 0−100; higher scores indicate better HRQoL.

A descriptive overview of the scale values for all time points, separated by group, is found in Table 8. A graph of the mean values for all measurement points, separated by group, is found in Figure 5. LMMs did not yield significant results for PedsQL scale. Relevant parameters (unstandardized regression coefficients with standard errors) are found in Table 5. The targeted effect size was not reached.

**Table 8:** Values for PedsQL total score separated by groups and measurement time points.

	PedsQL total score					
	IG			CG		
	T0	T1	T2	T0	T1	T2
*n*	19	15	19	10	11	11
M (SD)	45.01 (12.88)	45.47 (14.49)	44.08 (15.69)	42.23 (14.03)	43.02 (8.50)	12.41 (23.33)
min	25.00	25.00	25.00	27.08	30.00	23.33
max	64.58	68.75	75.00	65.38	56.67	60.00

## 4. Discussion

This study evaluated a CM intervention for its effects on care integration within the care network of children with SMA I and II as well as on caregivers’ and children's HRQoL. We designed the CM intervention to assess the current care situation with families, identify individual needs, and provide necessary information. Therefore, the case managers conducted regular, structured conversations with caregivers regarding the overall care focusing on supporting families in care integration. We implemented semi-structured interviews and questionnaires at three measurement points (T0–T2) to yield chosen outcomes in IG and CG. The IG caregivers described using CM for coordinating appointments (especially within the neuromuscular center), organizing assistance (e.g., for aids or prescriptions), and as a source of information. Furthermore, it provided support regarding contact referrals and in communicating with healthcare professionals. In addition, CM served an accompaniment function in that the caregivers regularly discussed the child's care status and well-being, and the entire family's needs with the case manager. The caregivers agreed individually on the frequency and type of contact.

The CM intervention seemed to have some influence on caregiver-reported care integration. Although the effect size of the PICS-D scale “Team quality & communication” is within our targeted range, Figure 2 (left side) indicated that IG participants reported better collaboration in the care network than did CG caregivers at all measurement time points, even at T0 (start of intervention). We assume the detected effect is more likely a potential effect of the different care context than of the CM intervention because we had assigned participants to groups according to the neuromuscular center they were registered at (IG = neuromuscular center in Freiburg; CG = neuromuscular center in Tuebingen/Essen). In the interviews, many IG caregivers confirmed this assumption. They reported that CM's outreach was too limited and did not affect their broader care network. It would mainly be effective within the structures of the Freiburg area's neuromuscular center (e.g., arrangements with other departments within the hospital). Families whose care network was farther away reported that the CM intervention did not support information exchanges between healthcare providers in and outside the hospital. Furthermore, some IG caregivers stated that their care network was already well established and/or they had already had contact persons at other institutions. They reported that the CM intervention had little influence on these consolidated structures.

Nonetheless, CM did seem to affect a key subset of care integration. Many IG caregivers reported that having a continuous point of contact led to better care coordination. According to the feedback in the interviews, the CM intervention reduces the workload by coordinating appointments and taking over bureaucratic tasks (e.g., communication with health insurance companies and authorities). In comparison, CG participants described having many contact points that they had to connect with individually. Some CG caregivers reported informal and unstructured care coordination support depending on the engagement in their care networks. The majority of IG caregivers experienced more systematic care, particularly during periods of new admissions and changes in the regularity of inpatient stays at T2. The case manager combined appointments efficiently, whereas CG participants reported single appointments demanding multiple visits to the neuromuscular center. Furthermore, CM seemed to be able to buffer the uncertainty caused by a potential switch to a new medication as well as strengthen the caregivers in their care management regarding the new challenges. The relief provided by the case manager's taking over of tasks is not (yet?) reflected in the quantitative outcomes. IG participants revealed relatively constant values on the stress/family impact scales (PICS-D scale “Family impact”, FaBel total score). Other factors could play a role here, e.g., the personality of the caregivers interviewed (Do I feel relieved?); the willingness to hand over tasks, as well as the child's care situation (Is there any task I could be relieved of right now, or are there tasks I could delegate?).

The CM intervention seemed to affect the caregivers’ and children's HRQoL marginally. The effect sizes for the SF-12 as well as PedsQL scales are in the lower range. Figures 4, 5 show no significant differences between CG and IG means across all measurement time points. We assume that a 12-month observation period is perhaps too short to detect significant effects of the CM intervention on HRQoL. Caregivers might notice effects on care integration (e.g., better care coordination through more efficient appointment coordination) faster because those effects are directly linked to their everyday care management. HRQoL is a dynamic concept that underlies the influence of many external factors (47). The caregivers provided information on potential factors that may have influenced their HRQoL in the interviews. COVID-19 caused a major disruption in the daily lives of families with children with chronic complex conditions. In addition, there have been major changes in the care dynamics of SMA through the approval of new drugs. These brought new challenges, uncertainties, and changes in the structure of care. In Figure 4 (left side), we observe a drop in both groups’ mean scores on the SF-12 scale “Mental Health” at T2. A lower drop in the IG may be associated with their interview statements that the CM intervention was able to support them in the uncertainty regarding the change to a new medication. Even if CM had little impact on HRQoL according to the quantitative outcomes, the interviews allow us to assume an outlook on the caregivers’ HRQoL. IG interview participants reported that CM provided a sense of security and continuity even when they did not use it frequently. They also felt that the case manager relieved them of tasks in organizing care. This fact might imply that had we evaluated the CM intervention over a longer period, HRQoL may have been positively affected. Regarding the influence on children's HRQoL, we additionally recommend the use of a more suitable questionnaire if the sample includes wheelchair-bound children. The PedsQL seemed to be insufficiently inclusive because of some items.

To our knowledge, this is the first study to have developed and examined a care coordination model in the context of SMA. We chose a patient-centered approach to optimally tailor the CM intervention to the needs of affected families. We previously conducted interviews with caregivers to assess the status of care, learn about families’ expectations, and consider disease-specific characteristics in the care organization [multi-perspective state analysis; (17)]. In two symposia, we finalized the CM intervention together with caregivers, chairpersons of patient advocacy organizations, and healthcare professionals involved in the care network. We applied both qualitative and quantitative methods to evaluate the CM intervention, which allowed us to depict individual perceptions, capture CM in its heterogeneity, and still elicit common components essential for a care coordination model. Furthermore, we inquired about suggestions for improvements to CM, creating a basis for designing future care models that can be also tailored to other pediatric chronic conditions.

This study describes a pilot evaluation of a CM intervention in children with SMA. Therefore, some aspects are limited and need future research. As SMA is such a rare disease, we could only recruit 32 participants despite an intensive recruitment strategy and support from our collaborative partners. Our sample in general is too small to detect group differences in quantitative outcomes according to our targeted effect sizes. As already mentioned, we divided the participants according to their affiliation with the neuromuscular centers, which is why the CG was smaller than the IG. Furthermore, participants received different incentives according to their group assignment. The different monetary amounts did not result arbitrarily from affiliation to the different neuromuscular centers but were deliberately chosen according to the different care models used in the study. In our view, CG participants should receive a higher incentive because IG participants benefited from the 1:1 care provision through the case manager. Moreover, caregivers reported heterogeneous care in the interviews depending on the neuromuscular center, family's place of residence, and more. However, we were not able to systematically record this heterogeneity within the scope of the study, so that we would have been able to make more concrete statements about CG's care, for example. Even though we failed to achieve the targeted effect sizes, note that the IG's mean scores for all quantitative measures are “better” than the CG's mean scores. Before the introduction of the CM intervention, a study assistant supported the IG caregivers in some aspects of care integration (e.g., appointment coordination in the neuromuscular center). Defining a general standard of “usual care” and comparing it to new forms of care coordination seemed difficult. We assume that the reported differences could have had an impact on the results of this study and might lead to a potential underestimation of CM effects. Therefore, we are not able to make causal, quantitative statements about a potential CM superiority compared with “usual care” in the context of this study. A proof of efficacy based on statistical significance tests was not this study's primary objective. Its focus is on assessing the feasibility and acceptability of CM as reported by IG participants because the qualitative results are able to better represent the heterogeneous evaluation of CM. Nonetheless, caregivers in both groups indicated a desire for better-integrated care, which, according to IG feedback, could be provided at least to some extent by CM.

In addition, the evaluation interval might have been too short because caregivers reported little change across measurement time points. We received feedback that the CM intervention was too abstract, theoretical, and difficult to integrate into one's own care management. Some caregivers reported in the T0/T1 interviews that they were initially unclear about what they could/did use CM for, and that many things had to settle in first. Since we developed the CM intervention at the beginning of the study, we assume that this could be a “start-up effect” that might be balanced if the CM intervention was evaluated over a longer period.

The CM concept developed for this study has been based on a CM model applied in other areas of the German healthcare system and tailored to the specific needs of families with a child diagnosed with SMA. Nonetheless, recent studies have found that the burdens experienced by caregivers of children with SMA are similar across countries (15, 17, 48). Even though some aspects may differ in other countries’ healthcare contexts, implementing care integration models comparable to CM might be essential across all contexts. As a result, different models emerged in other countries, e.g., Nurse Coaches in the US who also play an important role in coordinating interdisciplinary care teams (49). In most cases nurses and social workers choose to specialize in the field of care integration. They combine their clinical experience with coaching competencies to help patients and families enhance their disease management as it was the case in our study. As a future research topic, it would be interesting to compare different models and their respective training programs on an international level.

## 5. Conclusion

This study has provided important information on the development of CM from the perspective of caregivers of children with SMA I and II. We successfully determined that such a coordination model requires central components, e.g., structured assessments of the care situation and family's needs, appointment coordination, and discussion of care options. Nevertheless, it is also an individual “contract” between families and case managers to ensure individualized care. The CM intervention we developed was not equally helpful for all families. Many caregivers interviewed stick to their routine in their care management and were therefore unwilling or unable to delegate certain tasks. However, without exception, all families interviewed supported the existence of such a care model. The caregivers reported that they would rate CM as (even) more helpful earlier in the process. Therefore, we recommend a CM intervention immediately after diagnosis. Because caregivers of children with chronic conditions face similar demands and stresses, elements of our CM intervention may be applicable to other pediatric chronic conditions. Our study may support the further development of CM interventions that can be customized for specific diseases and implemented in regular care. Future multicenter studies investigating CM interventions at different sites are needed to reach more families and to depict broader care nationwide. They should include the perspectives of other stakeholders involved in care coordination (e.g., healthcare professionals). Further research should also relate the economic impact of CM interventions to the cost of regular care, e.g., the number of trips/time spent receiving services and lost work days. Because of the new drug therapies developing during our study, it would also be interesting to systematically compare groups based on the type of therapy received to see if it had an impact on the case management's assessment. Moreover, CM elements need to be adjusted according to the wishes we assessed in our pilot evaluation to tailor the model even more intensively to the caregivers’ needs.

## Data Availability

The raw data supporting the conclusions of this article will be made available by the authors, without undue reservation.

## References

[1] Mercuri E Finkel RS Muntoni F Wirth B Montes J Main M Diagnosis and management of spinal muscular atrophy: part 1: recommendations for diagnosis, rehabilitation, orthopedic and nutritional care. Neuromuscul Disord. (2018) 28(2):103–15. 10.1016/j.nmd.2017.11.005 29290580

[2] Finkel RS Mercuri E Darras BT Connolly AM Kuntz NL Kirschner J Nusinersen versus sham control in infantile-onset spinal muscular atrophy. N Engl J Med. (2017) 377(18):1723–32. 10.1056/NEJMoa1702752 29091570

[3] Mendell JR Al-Zaidy S Shell R Arnold WD Rodino-Klapac LR Prior TW Single-dose gene-replacement therapy for spinal muscular atrophy. N Engl J Med. (2017) 377(18):1713–22. 10.1056/NEJMoa1706198 29091557

[4] Al-Zaidy S Pickard AS Kotha K Alfano LN Lowes L Paul G Health outcomes in spinal muscular atrophy type 1 following AVXS-101 gene replacement therapy. Pediatr Pulmonol. (2019) 54(2):179–85. 10.1002/ppul.24203 30548438 PMC6590370

[5] Dabbous O Maru B Jansen JP Lorenzi M Cloutier M Guérin A Survival, motor function, and motor milestones: comparison of AVXS-101 relative to nusinersen for the treatment of infants with spinal muscular atrophy type 1. Adv Ther. (2019) 36(5):1164–76. 10.1007/s12325-019-00923-8 30879249 PMC6824368

[6] Baranello G Darras BT Day JW Deconinck N Klein A Masson R Risdiplam in type 1 spinal muscular atrophy. N Engl J Med. (2021) 384(10):915–23. 10.1056/NEJMoa2009965 33626251

[7] Dhillon S. Risdiplam: first approval. Drugs. (2020) 80(17):1853–8. 10.1007/s40265-020-01410-z 33044711

[8] Borell S Pechmann A Kirschner J. Spinale muskelatrophie. Monatsschr Kinderheilkd. (2015) 163(12):1293–304. 10.1007/s00112-015-0004-8

[9] Yeo CJJ Darras BT. Overturning the paradigm of spinal muscular atrophy as just a motor neuron disease. Pediatr Neurol. (2020) 109:12–9. 10.1016/j.pediatrneurol.2020.01.003 32409122

[10] Kuo DZ McAllister JW Rossignol L Turchi RM Stille CJ. Care coordination for children with medical complexity: whose care is it, anyway? Pediatrics. (2018) 141(Suppl 3):S224–32. 10.1542/peds.2017-1284G 29496973

[11] Hjorth E Kreicbergs U Sejersen T Lövgren M. Parents’ advice to healthcare professionals working with children who have spinal muscular atrophy. Eur J Paediatr Neurol. (2018) 22(1):128–34. 10.1016/j.ejpn.2017.10.008 29146237

[12] Altman L Zurynski Y Breen C Hoffmann T Woolfenden S. A qualitative study of health care providers’ perceptions and experiences of working together to care for children with medical complexity (CMC). BMC Health Serv Res. (2018) 18(1):70. 10.1186/s12913-018-2857-8 29386026 PMC5793356

[13] Finkel RS Mercuri E Meyer OH Simonds AK Schroth MK Graham RJ Diagnosis and management of spinal muscular atrophy: part 2: pulmonary and acute care; medications, supplements and immunizations; other organ systems; and ethics. Neuromuscul Disord. (2018) 28(3):197–207. 10.1016/j.nmd.2017.11.004 29305137

[14] Groen EJN Talbot K Gillingwater TH. Advances in therapy for spinal muscular atrophy: promises and challenges. Nat Rev Neurol. (2018) 14(4):214–24. 10.1038/nrneurol.2018.4 29422644

[15] Aranda-Reneo I Peña-Longobardo LM Oliva-Moreno J Litzkendorf S Durand-Zaleski I Tizzano EF The burden of spinal muscular atrophy on informal caregivers. Int J Environ Res Public Health. (2020) 17(23):8989. 10.3390/ijerph17238989 33276656 PMC7730048

[16] McMillan HJ Gerber B Cowling T Khuu W Mayer M Wu JW Burden of spinal muscular atrophy (SMA) on patients and caregivers in Canada. J Neuromuscul Dis. (2021) 8(4):553–68. 10.3233/JND-200610 33749617 PMC8385498

[17] Brandt M Johannsen L Inhestern L Bergelt C. Parents as informal caregivers of children and adolescents with spinal muscular atrophy: a systematic review of quantitative and qualitative data on the psychosocial situation, caregiver burden, and family needs. Orphanet J Rare Dis. (2022) 17(1):274. 10.1186/s13023-022-02407-5 35854387 PMC9295422

[18] Landfeldt E Pechmann A McMillan HJ Lochmüller H Sejersen T. Costs of illness of spinal muscular atrophy: a systematic review. Appl Health Econ Health Policy. (2021) 19(4):501–20. 10.1007/s40258-020-00624-2 33576939 PMC8270802

[19] Willems J Bablok I Farin-Glattacker E Langer T. Barriers and facilitating factors of care coordination for children with spinal muscular atrophy type I and II from the caregivers’ perspective: an interview study. Orphanet J Rare Dis. (2023) 18(1):136. 10.1186/s13023-023-02739-w 37268965 PMC10239104

[20] Inhestern L Brandt M Driemeyer J Denecke J Johannsen J Bergelt C. Experiences of health care and psychosocial needs in parents of children with spinal muscular atrophy. Int J Environ Res Public Health. (2023) 20(7):5360. 10.3390/ijerph20075360 37047974 PMC10094281

[21] Kölbel H Modler L Blaschek A Schara-Schmidt U Vill K Schwartz O Parental burden and quality of life in 5q-SMA diagnosed by newborn screening. Children. (2022) 9(12):1829. 10.3390/children9121829 36553273 PMC9776462

[22] Parast L Burkhart Q Gidengil C Schneider EC Mangione-Smith R Casey Lion K Validation of new care coordination quality measures for children with medical complexity. Acad Pediatr. (2018) 18(5):581–8. 10.1016/j.acap.2018.03.006 29550397 PMC6152933

[23] Walton H Hudson E Simpson A Ramsay AIG Kai J Morris S Defining coordinated care for people with rare conditions: a scoping review. Int J Integr Care. (2020) 20(2):14. 10.5334/ijic.5464 32607101 PMC7319081

[24] Singer SJ Burgers J Friedberg M Rosenthal MB Leape L Schneider E. Defining and measuring integrated patient care: promoting the next frontier in health care delivery. Med Care Res Rev. (2011) 68(1):112–27. 10.1177/1077558710371485 20555018

[25] Treinat L. E-Health als brücke zwischen den leistungsträgern. In: Müller-Mielitz S Lux T, editors. E-Health-Ökonomie. Wiesbaden: Springer Gabler (2017). p. 297–304.

[26] Hjorth E Kreicbergs U Sejersen T Jeppesen J Werlauff U Rahbek J Bereaved parents more satisfied with the care given to their child with severe spinal muscular atrophy than nonbereaved. J Child Neurol. (2019) 34(2):104–12. 10.1177/0883073818811544 30518279

[27] Porz F. Verzahnung stationärer und ambulanter versorgung in der pädiatrie: case management. In: Gerber A Lauterbach KW, editors. Gesundheitsökonomie und pädiatrie. Stuttgart: Schattauer Verlag (2006). p. 115–22.

[28] Wendt WR. Case management im sozial- und gesundheitswesen: Eine einführung. Freiburg: Lambertus-Verlag (2018). 354 p.

[29] Porz F Podeswik A. Case management in der kinderund jugendmedizin. In: Brinkmann V, editor. Case management: organisationsentwicklung und change management in gesundheits- und sozialunternehmen. Wiesbaden: Gabler (2010). p. 239–58. Available at: 10.1007/978-3-8349-8589-7_10 (Cited Mar 6 2019).

[30] Simon TD Whitlock KB Haaland W Wright DR Zhou C Neff J Effectiveness of a comprehensive case management service for children with medical complexity. Pediatrics. (2017) 140(6). 10.1542/peds.2017-1641 29192004

[31] Löcherbach P. Effektivität und effzienz von case management sind belegt. Bonn: MVF (2010). 27ff.

[32] Gordils-Perez J Schneider SM Gabel M Trotter KJ. Oncology nurse navigation: development and implementation of a program at a comprehensive cancer center. Clin J Oncol Nurs. (2017) 21(5):581–8. 10.1188/17.CJON.581-588 28945718

[33] Rare Disease UK. Rare disease care coordination: delivering value, improving services. London: Rare Disease UK (2013).

[34] Willems J Farin-Glattacker E Langer T. Evaluation of a case management to support families with children diagnosed with spinal muscular atrophy—protocol of a controlled mixed-methods study. Front Pediatr. (2021) 9:801. 10.3389/fped.2021.614512 PMC8369478 34414138

[35] Wang CH Finkel RS Bertini ES Schroth M Simonds A Wong B Consensus statement for standard of care in spinal muscular atrophy. J Child Neurol. (2007) 22(8):1027–49. 10.1177/0883073807305788 17761659

[36] Ziniel SI Rosenberg HN Bach AM Singer SJ Antonelli RC. Validation of a parent-reported experience measure of integrated care. Pediatrics. (2016) 138(6). 10.1542/peds.2016-0676 27940672

[37] Ravens-Sieberer U Morfeld M Stein RE Jessop DJ Bullinger M Thyen U. The testing and validation of the German version of the impact on family scale in families with children with disabilities. Psychother Psychosom Med Psychol. (2001) 51(9–10):384–93. 10.1055/s-2001-16899 11533885

[38] Stein RE Riessman CK. The development of an impact-on-family scale: preliminary findings. Med Care. (1980) 18(4):465–72. 10.1097/00005650-198004000-00010 7401703

[39] Morfeld M Kirchberger I Bullinger M. SF-36. Fragebogen zum gesundheitszustand. Deutsche version des short form-36 health survey. Manual. 2., ergänzte und überarbeitete auflage. Göttingen: Hogrefe Verlag GmbH & Co. KG (2011).

[40] Helfferich C. Leitfaden- und experteninterviews. In: Baur N Blasius J, editors. Handbuch methoden der empirischen sozialforschung. Wiesbaden: Springer Fachmedien (2014). p. 559–74. Available at: 10.1007/978-3-531-18939-0_39 (Cited Oct 13 2021)

[41] Chan KS Mangione-Smith R Burwinkle TM Rosen M Varni JW. The PedsQL: reliability and validity of the short-form generic core scales and asthma module. Med Care. (2005) 43(3):256–65. 10.1097/00005650-200503000-00008 15725982

[42] R Core Team. R: a language and environment for statistical computing. Vienna, Austria: R Foundation for Statistical Computing (2020). Available at: https://www.R-project.org/.

[43] Nakagawa S Johnson PCD Schielzeth H. The coefficient of determination R2 and intra-class correlation coefficient from generalized linear mixed-effects models revisited and expanded. J R Soc Interface. (2017) 14(134):20170213. 10.1098/rsif.2017.0213 28904005 PMC5636267

[44] Bates D Mächler M Bolker B Walker S. Fitting linear mixed-effects models using lme4. J Stat Softw. (2015) 67:1–48. 10.18637/jss.v067.i01

[45] Kuznetsova A Brockhoff PB Christensen RHB. Lmertest package: tests in linear mixed effects models. J Stat Softw. (2017) 82:1–26. 10.18637/jss.v082.i13

[46] Kuckartz U. Qualitative inhaltsanalyse. Methoden, praxis, computerunterstützung. 4. überarbeitete edn. Weinheim Basel: Beltz Juventa (2018). 240 p.

[47] Güthlin C. Response shift: alte probleme der veränderungsmessung, neu angewendet auf gesundheitsbezogene lebensqualität. Z Psychosom Med Psychother. (2004) 13(4):165–74.

[48] Landfeldt E Abner S Pechmann A Sejersen T McMillan HJ Lochmüller H Caregiver burden of spinal muscular atrophy: a systematic review. PharmacoEconomics. (2023) 41(3):275–93. 10.1007/s40273-022-01197-9 36515815

[49] Nurse Coaching | Holistic Nursing. The Nurse Coach Collective. Available at: https://thenursecoaches.com/ (Cited Jun 13 2023).

